# Sacral Osteomyelitis With Abscesses and Fistulae: An Unusual Manifestation of Disseminated Blastomycosis

**DOI:** 10.7759/cureus.95247

**Published:** 2025-10-23

**Authors:** Jose T Rodriguez, Andrew B Trotter

**Affiliations:** 1 Infectious Diseases, Universidad Iberoamericana, Santo Domingo, DOM; 2 Infectious Diseases, University of Illinois Chicago, Chicago, USA

**Keywords:** blastomycosis, disseminated fungal infection, epidural abscess, fistula, sacral osteomyelitis

## Abstract

*Blastomyces dermatitidis*, the fungus responsible for blastomycosis, can cause systemic disease with clinical manifestations ranging from asymptomatic infection to acute or chronic pneumonia and extrapulmonary diseases. Here, we present a case of sacral osteomyelitis with multiple abscesses and a fistulous tract, which failed to improve after multiple courses of antibacterial therapy for presumed bacterial causes, leading to a significantly delayed diagnosis. Blastomycosis was diagnosed by potassium hydroxide (KOH) microscopy, fungal culture, and broad-range polymerase chain reaction (PCR). The patient was prescribed oral itraconazole, 200 mg three times a day for three days, followed by twice a day for one year.

The presentation of abscesses and osteomyelitis with a fistulous tract in blastomycosis is uncommon, and one of the rare instances of dissemination and destruction of the sacral bone is being reported. The key to diagnosing the disease is considering it a possibility. Treatment depends on its severity.

## Introduction

Blastomycosis is a fungal disease caused by thermally dimorphic fungi in the genus *Blastomyces*, with *B. dermatitidis *complex responsible for most cases [1]. This organism is primarily found in regions of the Ohio and Mississippi River Valleys, the Great Lakes, and parts of Canada. Infection usually occurs through the inhalation of conidia, leading to pulmonary infection [2]. The spectrum of clinical presentation of blastomycosis is heterogeneous, ranging from asymptomatic infection to fulminant disease with multisystem organ failure, depending on the extent of exposure and host immune status [1].

Dissemination is common in 25% to 40% of all symptomatic infections. *B. dermatitidis* can infect nearly every organ, with the most common extrapulmonary sites being the skin, bone, genitourinary tract, and central nervous system (CNS). Bone or joint involvement is present in 5% to 20% of patients with disseminated blastomycosis. CNS involvement is present in 5% to 10% of immunocompetent patients with disseminated blastomycosis [1].

The key to establishing a diagnosis of blastomycosis is considering this disease as a possibility. Because blastomyces is acquired from the environment, a history of the patient’s residence and travel should be obtained, with attention to possible exposure to moist soil, lakes, or work in excavations or construction, as these are areas where the organism can be commonly found. The definitive diagnosis of blastomycosis is made by isolating *B. dermatitidis* in culture. However, molecular methods, such as polymerase chain reaction (PCR) assays and next-generation sequencing, are increasingly used, as they provide faster and more sensitive detection than culture. In general, treatment is similar for both pulmonary and disseminated infection; the severity of the illness directs the medication strategies. Milder infections are treated with oral azole agents, and severe infections require initial therapy with amphotericin B [3].

## Case presentation

A 29-year-old female from Illinois with a significant past medical history of splenectomy for splenic abscesses complicated by coloenteric fistula (status post left colectomy) and pelvic inflammatory disease (pyosalpinx and tubo-ovarian abscess) was transferred to our center for escalation of care after several months of multiple antibacterial trials with no improvement and worsening abscesses and sacral osteomyelitis identified by imaging. Prior to transfer, she had completed four days of piperacillin-tazobactam therapy.

Upon arrival, the patient reported three days of nausea, vomiting, diarrhea, and flank pain and was unable to tolerate oral consumption. Her vital signs were stable, and there were no signs of meningeal irritation or sacral pain during palpation. Given her persistent symptoms and previous imaging from other institutions concerning sacral osteomyelitis and epidural abscess, further evaluation was performed to assess possible disease dissemination. 

**Figure 1:**
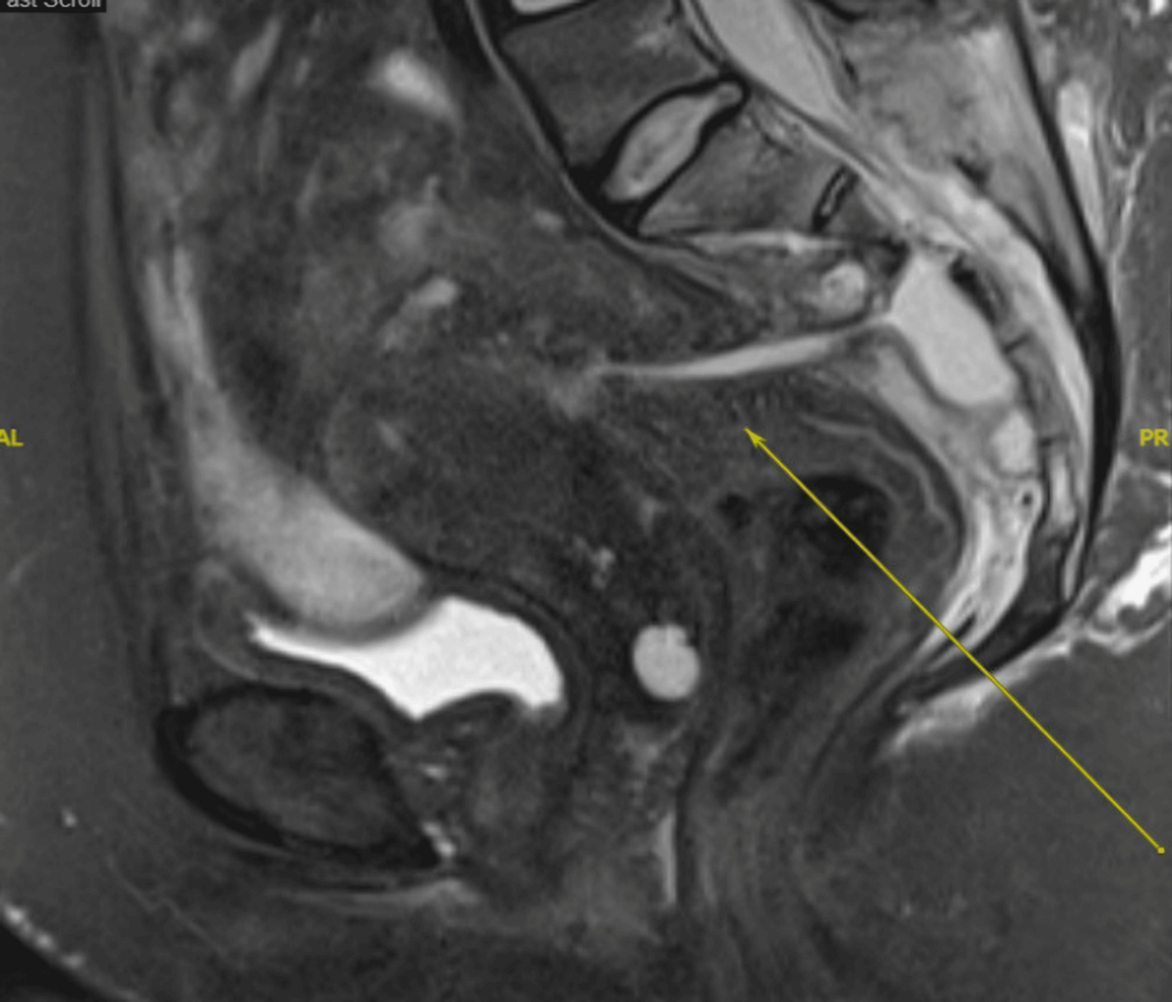
MRI with contrast: Fistulous tract extending from the superior medial aspect of the collection towards the fluid collection in the sacrum. The sacral collection extends posteriorly into the sacral spinal canal via an eroded defect in the sacrum, impressing on the thecal sac.

**Figure 2:**
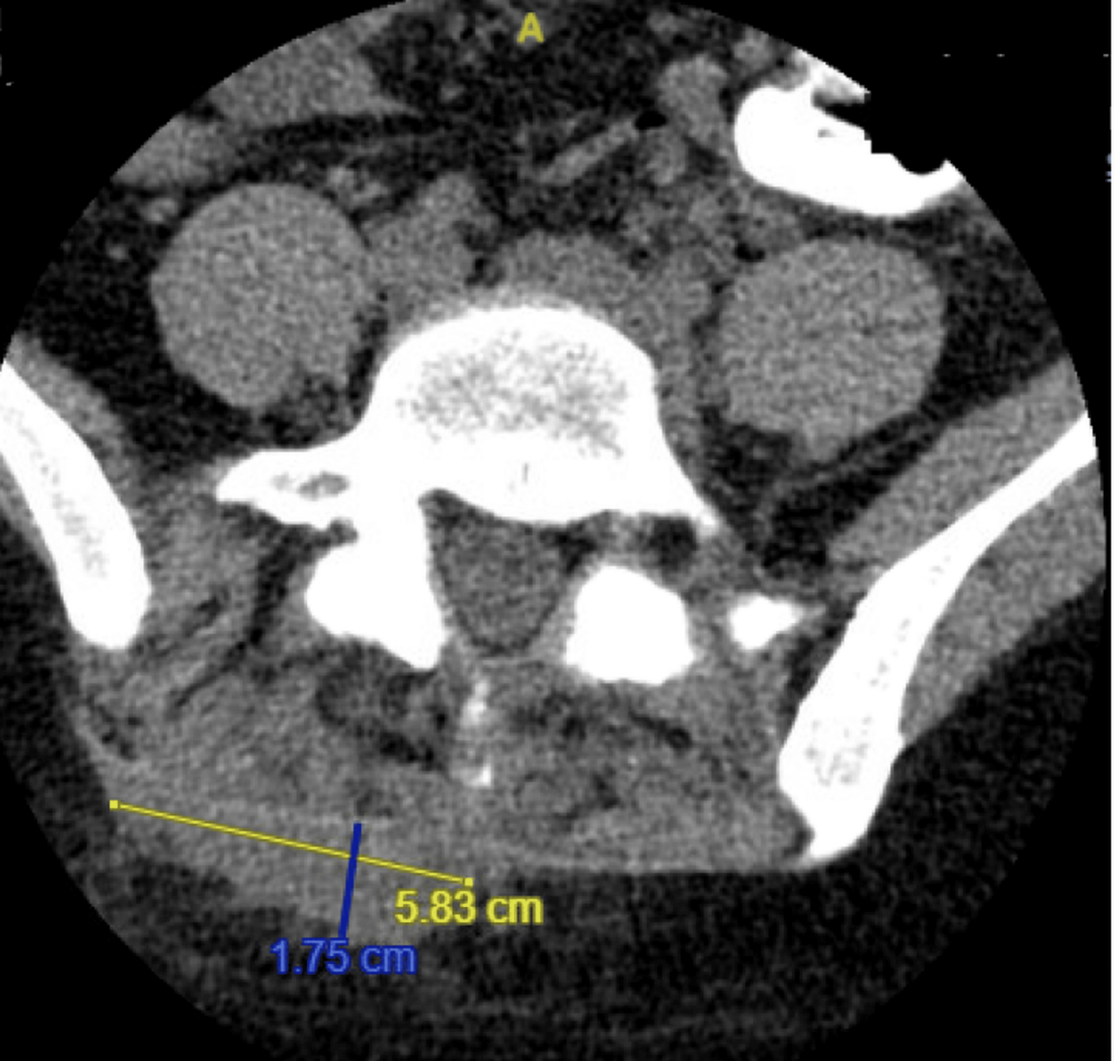
Non-contrast CT of the lumbar spine demonstrates a complex subcutaneous abscess (1.8 cm anterior x 5.8 cm transverse x 5.6 cm craniocaudal) surrounding inflammatory stranding.

**Figure 3:**
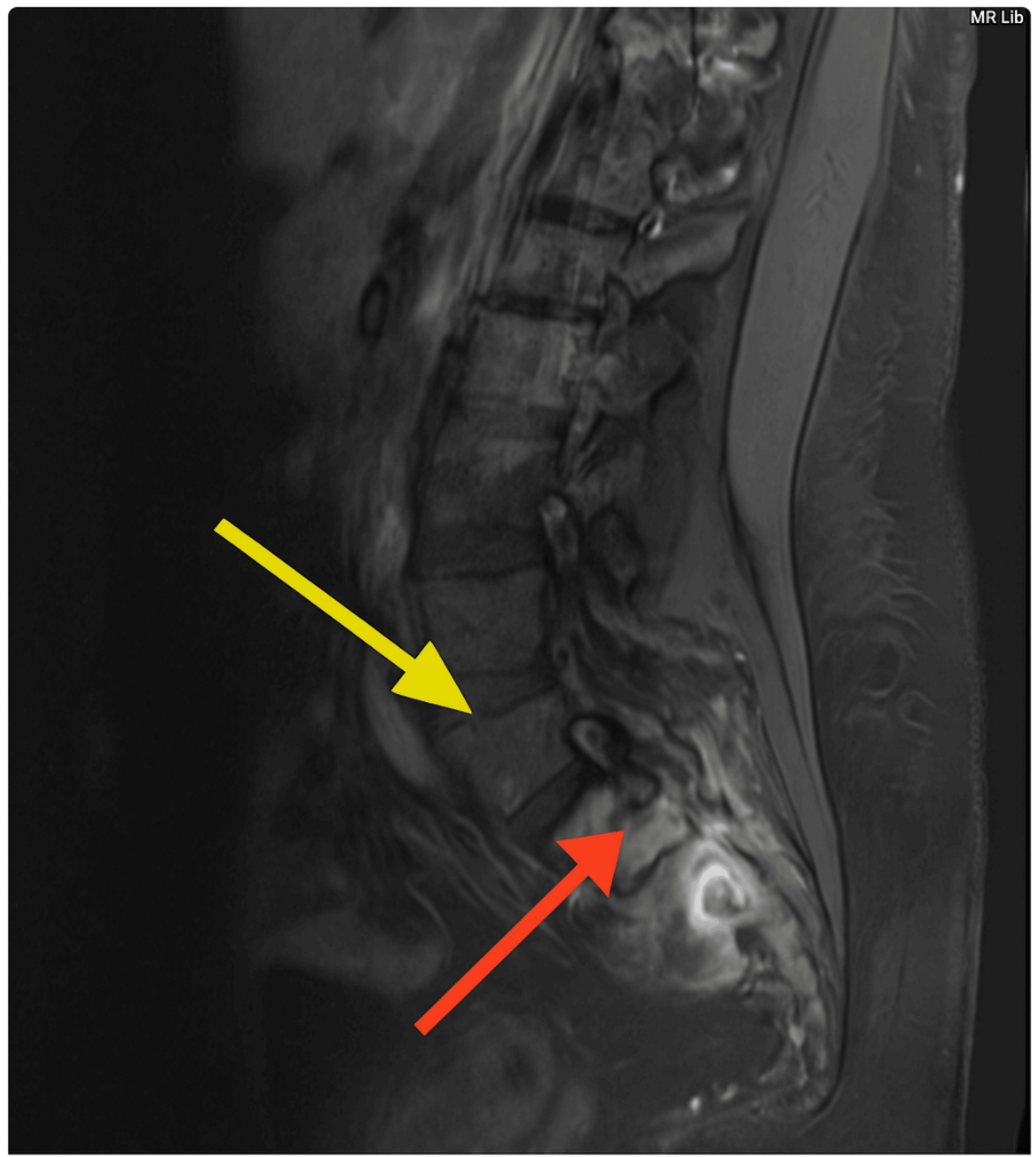
MRI without contrast revealed erosion of the S1 spinous processes (yellow arrow) and fluid collection adjacent to inflammatory changes consistent with abscess formation (red arrow) and osteomyelitis.

MRI with contrast demonstrated presacral and right posterior lower lumbosacral paraspinal abscesses with fistulous extension, as shown in Figure 1. A non-contrast CT revealed paraspinal abscess formation and soft tissue thickening around an eroded S1 area, suggestive of osteomyelitis, with spinal canal indentation at the sacral level, indicating possible epidural extension, as shown in Figure 2. MRI without contrast (Figure 3) demonstrated erosion of the S1 spinous process with adjacent fluid collection and inflammatory changes and acute osteomyelitis involving the sacrum and enhancement with edema in bilateral iliac bones (likely reactive).

Given the presence of an epidural abscess, antibiotics were changed from piperacillin-tazobactam to vancomycin, cefepime, and metronidazole for better CNS penetration. Neurosurgery, Interventional Radiology (IR), and Gastrointestinal were consulted for consideration of surgical intervention, biopsy, and culture. Neurosurgery deferred surgical intervention, IR performed a CT-guided aspiration of the L5 posterior paraspinal fluid collection, yielding four cc of blood-tinged purulent fluid that was sent for bacterial and fungal cultures, and Gastroenterology ruled out inflammatory bowel disease (IBD) through flexible sigmoidoscopy, which revealed a benign-appearing area of anastomosis or intrinsic stenosis measuring 4 cm (in length) x 8 mm (inner diameter) at 20 cm proximal to the anus. 

Despite empiric broad-spectrum antibiotic therapy, the patient's condition did not improve. A transthoracic echocardiogram (TTE) was requested to evaluate a possible hematogenous source, which showed a normal left ventricular ejection fraction, no evidence of vegetations, and no significant valvular regurgitant issues. A bone biopsy was required to assess the acute osteomyelitis and culture for bacterial or fungal causes.

On hospital day fourth of empiric antibiotic treatment, the patient developed a rise in creatinine from 0.69 mg/dL at admission to 2.17 mg/dL, which prompted a change from vancomycin to daptomycin. After five days of empirical antibiotic therapy, cultures of the abscesses and blood continued to yield negative results, and despite achieving therapeutic drug levels, the WBC count rose to 21.7 K/uL, with a predominance of neutrophils. Creatinine was 1.90, and therapeutic levels were achieved on the medication. 

A fungal or aseptic cause was suspected for this disease progression despite persistently negative cultures. A temporary hold on the antibiotics was recommended on this hemodynamically stable patient till obtaining a sacral biopsy to guide medication therapy. After seven days, without progression of the disease, an immunodeficiency workup was sent, and HIV testing was negative.

The patient remained off of antibacterial coverage until IR performed a second aspiration of lumbar fluid collection and sacral bone biopsy. The sample was sent for evaluation of bacterial, fungal, acid-fast bacilli, and histopathology for actinomycosis. The specimen was also sent to the University of Washington for broad-range PCR.

The laboratory specimen cultures yielded a few broad-based yeasts consistent with *Blastomyces* *gilchristii*. The recommendations for 200 mg of oral itraconazole, three times a day for three days, followed by two times a day for a year, weekly liver function tests, a visit to the infectious disease clinic in a month for follow-up, and chest x-rays to rule out disease dissemination, were made, with follow-up histopathology of the University of Washington PCR and continuing workup to rule out immunodeficiencies.

Laboratory findings on arrival and admission were relevant for a high white blood cell count, suggesting an ongoing inflammatory process. Urinalysis demonstrated hematuria and mild proteinuria, without bacteriuria, raising concern that the urinary abnormalities were secondary to a systemic illness rather than a primary urinary tract infection, with no bacterial presence, as illustrated in Table 1. Procalcitonin was obtained once and was found to be low, suggesting that severe bacterial infection was less likely, as illustrated in Table 2. Physical examination was unremarkable except for flank pain.

**Table 1:** Laboratory results during hospital admission. WBC: white blood cells, BUN: blood urea nitrogen, eGFR: estimated glomerular filtration rate

Test	First	Second	Fourth	Fifth	Eighth	Seventeenth	Reference Range
WBC (10³/µL)	17.4 (10³/µL)	18.8 (10³/µL)	19.5(10³/µL)	21.7 (10³/µL)	20.1(10³/µL)	18.0 (10³/µL)	4.0-11.0 (10³/µL)
BUN (mg/dL)	6 mg/dL	5 mg/dL	16 mg/dL	18 mg/dL	13 mg/dL	6 mg/dL	7-20 mg/dL
Creatinine (mg/dL)	0.73 mg/dL	0.69 mg/dL	2.17 mg/dL	2.30 mg/dL	1.48 mg/dL	0.84 mg/dL	0.6 – 1.2 mg/dL
eGFR (mL/min/1.73 m²)	117 mL/min/1.73 m²	121 mL/min/1.73 m²	31 mL/min/1.73 m²	29 mL/min/1.73 m²	49 mL/min/1.73 m²	97 mL/min/1.73 m²	>90 mL/min/1.73 m²

**Table 2:** Procaltonin value at admission.

Test	Value	Reference Range
Procalcitonin (ng/mL)	0.13	<0.25

## Discussion

Blastomycosis is a systemic fungal infection caused by *Blastomyces dermatitidis* and *Blastomyces gilchristii*, typically affecting the lungs and, in disseminated cases, the skin, bones, and genitourinary system. The disease can present with a broad spectrum of clinical manifestations, often mimicking bacterial infections or malignancies, which may delay diagnosis. Disseminated disease occurs in approximately 25-50% of symptomatic cases, most often involving the skin and bones [1,3].

We presented a case of a young woman with an atypical presentation of disseminated blastomycosis involving sacral destruction, fistulae, and multiple abscesses. Notably, she had no respiratory symptoms, no abnormalities in chest imaging, cutaneous lesions suspicious for blastomycosis or trauma that could suggest direct inoculation of the fungus, and negative cultures. 

Notably, the isolate in this case was identified as *Blastomyces gilchristii*, which is a recently described cryptic species within the *B. dermatitidis* complex [4]. The geographic distribution appears to be more restricted to areas surrounding the Great Lakes and parts of Canada, where it has been implicated in outbreak investigations [4,5]. Clinical manifestations are broadly similar to those of *B. dermatitidis*, although *B. gilchristii* has been associated with a higher proportion of pulmonary disease [5]. Traditional culture methods do not differentiate these species; molecular techniques are required. Including the species designation adds epidemiologic precision and clinical relevance, as *B. gilchristii* remains relatively uncommon in published clinical reports.

*Blastomyces* spp. are thermally dimorphic fungi that cause pyogranulomatous infection. Inhaled spores of the blastomyces could lead to an infection of the lungs that can progress from an asymptomatic disease to severe, life-threatening complications. Disseminated blastomycosis can occur via lymphohematogenous spread to the skin, bones, joints, genitourinary tract, and central nervous system [6]. Extrapulmonary infection with *B. dermatitidis* is commonly seen as verrucous skin lesions. CNS blastomycosis remains a rare manifestation, occurring in only 5-10% of cases of disseminated disease [7].

The bone is the second most common site of dissemination. Long bones, ribs, skull, and lower thoracic and lumbar spines osteomyelitis has been more frequently reported with blastomycosis, although essentially any bone can be affected. Fungal infections of the spine are uncommon but not rare; delays in diagnosis are common. Frazier et al. described a three-month delay in diagnosis of fungal infection in a retrospective review [7,8].

Importantly, no physical findings or imaging features are diagnostic for blastomycosis, diagnosis requires microbiologic or histopathologic confirmation, such as culture or direct visualization of the organism. The differential diagnosis of blastomyces dermatitidis is broad and includes noninfectious diseases such as sarcoidosis and metastatic cancer, and various infectious etiologies. Blastomycosis can affect any organ, most commonly presenting as pneumonia, cutaneous ulcers, or verrucous skin lesions; however, the diagnosis is often delayed. The disease is frequently misdiagnosed, particularly as malignancy [9,10]. Cases reporting mandibular osteomyelitis and the use of prolonged antibiotics failing to improve symptoms have been widely documented [6].

There have been cases where patients were diagnosed with blastomycosis mimicking tuberculosis and treated actively for tuberculosis infections for six months, leading to a delay in diagnosis and further complications [11,12]. Blastomycosis can produce clinical findings or symptoms that mimic bacterial sources, including mimicking bacterial cellulitis in which patients receiving antibiotics without any resolution of their symptoms also delays the diagnosis [13,14].

Reported cases of patients presenting with an incidental mass resembling head and neck cancer or breast malignancies that initially presented as infections, and patients receiving multiple trials of oral and topical antibiotics [15,16].

Early diagnosis and treatment improve prognosis [8]. In the case described, the diagnosis was made 17 days after the patient's initial presentation in our hospital. However, the patient was seen at multiple outside institutions and was ill for months prior to being seen in our institution, highlighting blastomycosis as a diagnostic challenge due to its atypical presentations.

Before initiating therapy, a comprehensive test is required to evaluate renal, hematologic, and hepatic function, as well as potential drug-to-drug interactions. This evaluation is particularly important for amphotericin B, which is associated with nephrotoxicity, while azoles such as itraconazole require hepatic monitoring for drug-drug interactions. After evaluation is finished, initiation of antifungal treatment to reduce fatality, which is around 4.3% and 6.3% in immunocompetent patients and nearly 40% in AIDS patients with most deaths occurring within three weeks of diagnosis [17]. 

## Conclusions

This case of disseminated blastomycosis presenting with sacral osteomyelitis with fistulae and abscesses in an immunocompetent patient. The absence of classic pulmonary or cutaneous involvement features made diagnosing it difficult. This case highlights the need to consider blastomycosis in the differential diagnosis of osteomyelitis with abscesses, even in the absence of pulmonary findings. 

Multidisciplinary collaboration is crucial for atypical osteolytic lesions and the presence of abscesses, as it facilitates diagnostic evaluation and prompt treatment. Particularly in endemic areas, and to initiate antifungal therapy promptly. These are essential to improving patient outcomes, reducing the risk of dissemination, and preventing life-threatening complications.
